# Impact of Phacoemulsification on Trabeculectomy Bleb Function and Morphology in Primary Angle Closure Glaucoma: A Comparative Study of the Visco-Cushion Effect

**DOI:** 10.7759/cureus.70749

**Published:** 2024-10-03

**Authors:** Kirti Singh, Keerti Wali, Arshi Singh, Mainak Bhattacharyya, Sonal Dangda

**Affiliations:** 1 Ophthalmology, Guru Nanak Eye Centre, Delhi, IND; 2 Ophthalmology, Shri B. M. Patil Medical College, Bijapur Lingayat Development Education (BLDE) (Deemed to be University), Vijayapura, IND; 3 Ophthalmology, EyeQ & Max Healthcare, Ghaziabad, IND; 4 Ophthalmology, Atrium Health Wake Forest Baptist, Winston-Salem, USA

**Keywords:** anterior segment optical coherence tomography (asoct), bleb failure, ibags, ophthalmic viscoelastic device, phacoemulsification, primary angle-closure glaucoma, trabeculectomy bleb

## Abstract

Purpose

This study evaluates the impact of phacoemulsification on trabeculectomy bleb morphology and intraocular pressure (IOP) control in patients with primary angle closure glaucoma (PACG). The study also evaluates possible alterations in these effects by intra-operative blocking of internal sclerostomy by high-density viscoelastic (visco-cushion).

Methods

This is a single-center, interventional, prospective study including patients with PACG who underwent phacoemulsification post-trabeculectomy. This study also evaluated the effect of sodium hyaluronate 1.4% as a cushion to block the sclerostomy site. Patients were divided into two groups: group A received this intervention, while group B did not. Postoperative IOP and the number of glaucoma medications at week 2, month 1, and month 4 were assessed as a measure of bleb function. Bleb morphology was analyzed at these timepoints using the Indiana Bleb Appearance Grading Scale (IBAGS) and anterior segment optical coherence tomography (AS-OCT).

Results

The study included 35 patients with a mean age of 59.91 ± 7.59 years. The mean interval between trabeculectomy and phacoemulsification was 6.83 ± 5.57 years (range: 1-20 years). Mean preoperative IOP was 15.43 ± 2.62 mm Hg, with 12 eyes needing anti-glaucoma medications (AGMs). Mean IOP at postoperative month 4 was 12.69 ± 2.32 mm Hg (p<0.001, chi-square test), with one eye needing AGM. IOP reduction was significantly lower in the visco-cushion group (p<0.05, ANOVA test). By the end of four months, 97.14% of patients showed complete success as compared with 65% preoperatively. Bleb morphology was noted to be maintained in up to 66% patients in terms of bleb height and extent on IBAGS, as well as bleb internal reflectivity and the number of microcysts, as noted on AS-OCT. The group without a visco-cushion exhibited a significant decrease in qualitative bleb height and microcystic spaces, along with an increase in bleb vascularity (p<0.05, Fischer exact test). Thirty patients (87.5%) showed a >20% decline in bleb height on AS-OCT, which was greater in group without visco-cushion (p<0.05, Mann-Whitney U test).

Conclusion

Use of a visco-cushion during phacoemulsification in PACG eyes with prior functioning trabeculectomy resulted in the retention of healthy bleb morphology parameters except bleb wall thickening. However, this protective effect on bleb morphology did not transcribe into IOP reduction. On the contrary, conventional phacoemulsification despite bleb height reduction, increased bleb vascularity, and decreased microcystic spaces resulted in better IOP control in the first four months after surgery. Longer follow-up of these cases is suggested to examine eventual fate of these bleb morphological alternations.

## Introduction

Progression of cataract is sometimes accelerated after trabeculectomy, and performing cataract surgery in eyes with functional blebs has the inherent risk of jeopardizing bleb function. This detrimental effect of cataract surgery on functioning bleb has been documented by many studies [1-6], with need for long-term need for anti-glaucoma medications (AGMs) to control intraocular pressure (IOP), whereas others have shown no significant effect [7-11]. Most of these studies have evaluated trabeculectomy in primary open angle glaucoma (POAG) eyes, with the literature being relatively silent for primary angle closure glaucoma (PACG). Anatomic factors dictate that PACG blebs would behave differently after removing one of the main etiologies of disease, namely bulky lens. To address this gap, this study was designed to evaluate impact of phacoemulsification in prior filtered PACG eyes, focusing on bleb morphology (both qualitatively and quantitatively) along with bleb function (IOP control).

Low-grade inflammation in the anterior chamber (AC) secondary to lens protein exposure [12] may last for six months after cataract surgery [13]. Effect of ultrasound energy, intra-operative AC turbulence, and postoperative AC inflammation are postulated to cause bleb failure post-phacoemulsification [14]. Blocking the sclerostomy during phacoemulsification could help prevent factors leading to bleb damage. We used a novel technique of blocking sclerostomy with high-density cohesive viscoelastic (sodium hyaluronate 1.4%) in one arm of our study, referred throughout the paper as “visco-cushion.”

## Materials and methods

Study design

This is a single-center prospective interventional study conducted at Guru Nanak Eye Centre, Delhi, India, between November 2014 and March 2017 to study the surgical outcome and impact of phacoemulsification on bleb function, performed with and without visco-cushion. The Institutional Review Board (IRB) of Maulana Azad Medical College granted the IRB approval for patient enrollment, data collection, and anterior segment optical coherence tomography (AS-OCT) imaging for the assessment of the bleb morphology.

Participants

We recruited patients with known PACG and visually significant cataracts, all of whom had undergone previous trabeculectomy. Bleb function was assessed by gonioscopy (patent sclerostomy) and good IOP control. A minimum time interval of six months was taken between trabeculectomy and phacoemulsification to allow for the bleb to mature structurally and functionally.

Patients with open angle glaucoma, patients with any form of secondary glaucoma such as uveitic, neovascular, lenticular, pigmentary, pseudo-exfoliation, traumatic, and developmental glaucoma, and patients with any other coexistent ocular disease or prior ocular surgery other than trabeculectomy were excluded from the study.

Sample size calculation

Using the mean change in IOP value of 2.79 ± 4.66 mm Hg as the reference [15], the minimum required sample size with 90% power and 5% level of significance was calculated to be 29. A total of 35 patients (single eye) were enrolled.

Randomization

To further assess the effect of blocking the internal sclerostomy by Healon GV® (sodium hyaluronate 1.4%), we employed computer generated randomization to divide the patients into two groups: group A underwent phacoemulsification with visco-cushion, and group B underwent phacoemulsification without visco-cushion. The allocation was concealed from the observer (A.S.) collecting the data by opaque sequentially numbered sealed envelopes. Informed consent was obtained from all patients after clearly explaining the prognosis, risks, benefits, and alternatives of the procedure in their local language.

Technique

Single surgeon (K. S.) performed all surgeries under a peribulbar block (5-7cc) consisting of 2% lidocaine and 0.75% bupivacaine. Under standard aseptic precautions, temporal clear corneal incision along with two paracentesis incisions were made, and dispersive viscoelastic device was injected to fill the AC. In cases with posterior synechiae and non-dilating pupil, synechiolysis was performed using a vitreous, sweep and iris hooks were used as needed. A continuous curvilinear capsulorrhexis of 6-7 mm was successfully attempted in all cases. Phacoemulsification was performed using the “stop and chop” technique under low fluidics, followed by placement of a single-piece posterior chamber intraocular lens in the capsular bag. Additionally, in group A, sodium hyaluronate 1.4% was injected at the internal ostium, i.e., the sclerostomy site, prior to capsulorrhexis and during the phacoemulsification procedure. Internal ostium was identified based on the location of iridectomy and passive staining by trypan blue dye injected for anterior capsule staining.

At the end of the procedure, a thorough removal of the viscoelastic was performed, and intracameral carbachol was injected to constrict the pupil. All incision wounds were hydrated, and AC was reformed with balanced salt solution. Subconjunctival injections of gentamycin (0.5 mL) and dexamethasone (0.5 mL) were given in the inferior fornix before patching the eye. Postoperative care consisted of topical moxifloxacin 0.5% four times a day for two weeks and topical 1% prednisolone acetate eight times a day for three days, which was tapered over six weeks. We monitored postoperative IOP and administered topical/systemic AGMs if IOP was above 18 mm Hg.

Assessment and outcomes

Detailed medical and surgical history was recorded for all cases. Preoperative baseline examination included uncorrected visual acuity, IOP, anterior segment assessment including gonioscopy and fundoscopy, automated Humphrey visual field test (where possible), and biometry by A-scan (Sonomed PacScan 300AP). Pre- and postoperative baseline examinations included visual acuity (Snellen’s chart), IOP (Goldmann’s applanation tonometry), and number of AGMs. Intra- and postoperative complications, if any, were noted for all patients. Preoperative data were collected one week prior to the procedure, and postoperative data were collected at two weeks, one month, and four months after surgery. Additional postoperative parameters included bleb morphology by slit-lamp examination for the Indiana Bleb Appearance Grading Scale (IBAGS) [16] grading (height, extent, vascularity, and leakage) and AS-OCT (RTVue, Optoview Inc., Fremont, CA), To remove observer bias, two different trained observers evaluated the bleb using IBAGS (K.W. ) and AS-OCT (M.B.).

**Figure 1:**
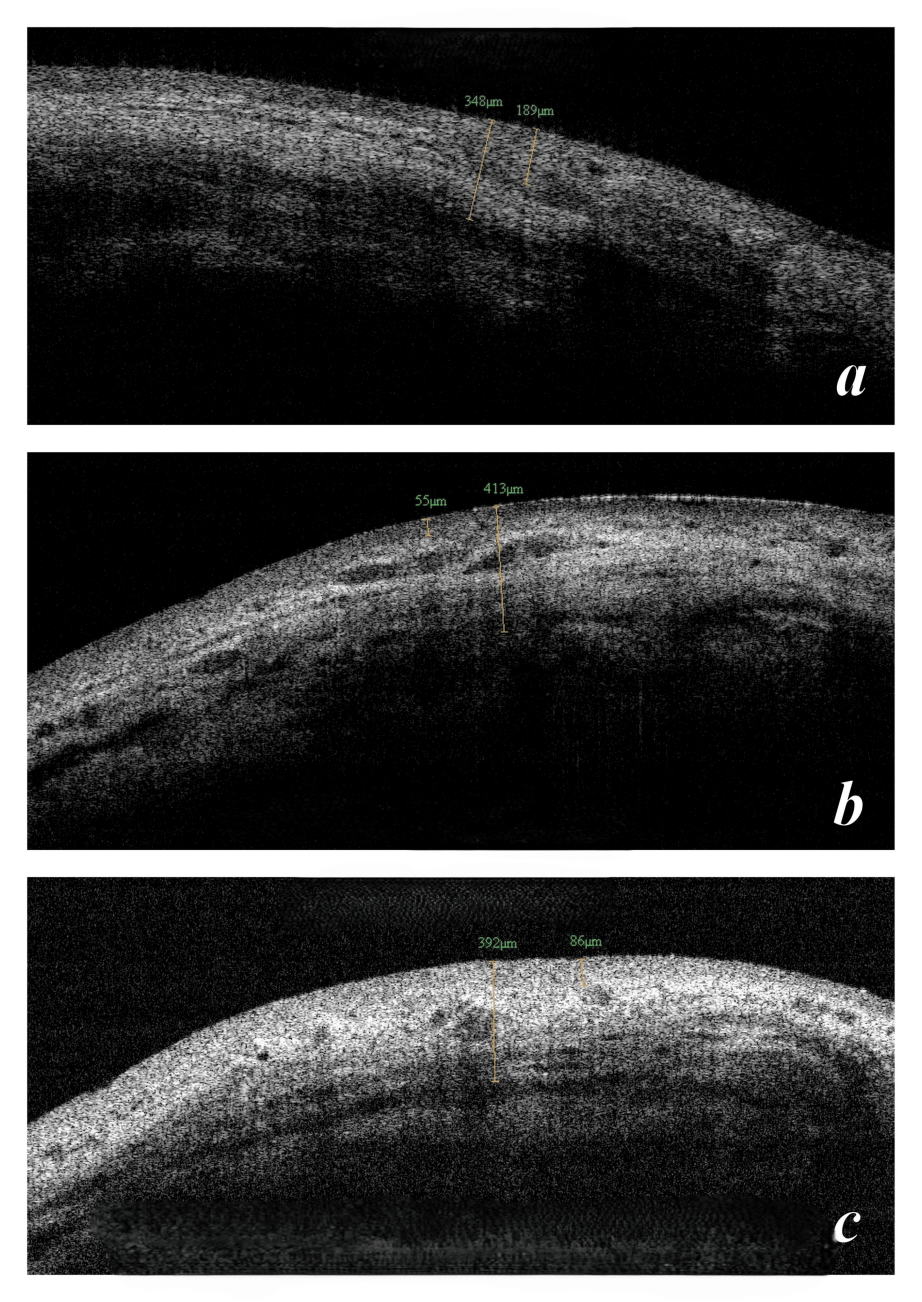
Vignette of internal reflectivity grading on AS-OCT Figure 1a shows low internal reflectivity graded as 1, Figure 1b shows moderate internal reflectivity graded as 2, and Figure 1c shows high internal reflectivity graded as 3 on the Likert scale. AS-OCT, anterior segment optical coherence tomography

**Figure 2:**
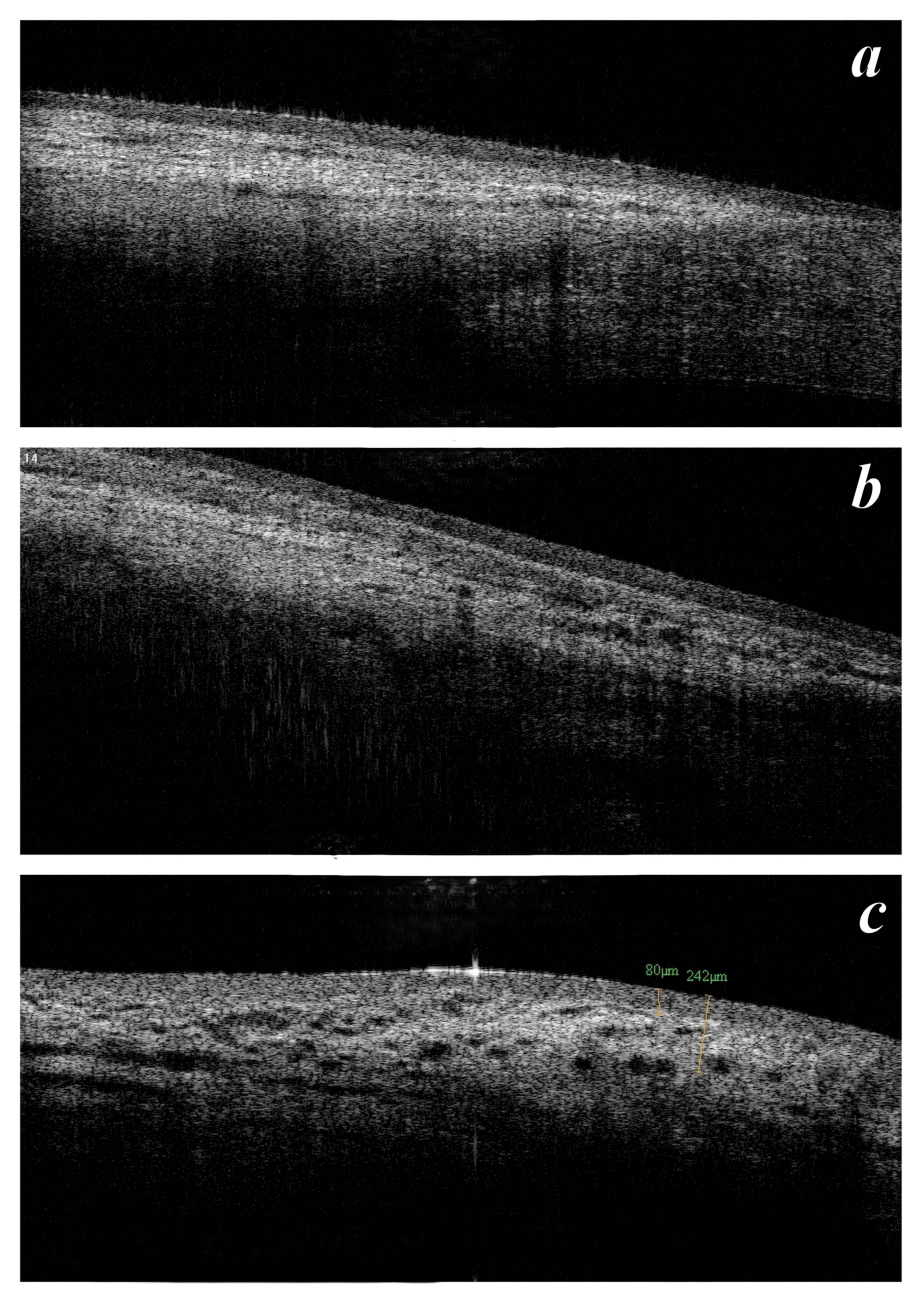
Vignette of microcystic spaces grading on AS-OCT Figure 2a shows minimal microcystic spaces graded as 3, Figure 2b shows a moderate number of microcystic spaces graded as 2, and Figure 2c shows a large number of microcystic spaces graded as 1 on the Likert scale. AS-OCT, anterior segment optical coherence tomography

To obtain the AS-OCT, the patients were instructed to look down and the upper lid was manually retracted to expose the conjunctival filtering bleb superiorly. Multiple vertical 2-dimensional images were obtained using the raster program. The shape of the blebs was noted as either cystic, flat, or encapsulated. Bleb height was measured from the scleral hyperreflective margin to the superior extent of the bleb including the bleb wall. Bleb wall thickness was measured at the 12 O'clock position, at the area of maximum thickness, and at sclerostomy site if possible. Bleb reflectivity throughout the bleb area was noted along with evidence of microcystic/macrocystic spaces. An objective evaluation of bleb internal reflectivity was performed and categorized as per descending Likert scale: 1, low reflectivity (good bleb); 2, moderate hyperreflectivity; and 3, severe hyperreflectivity (poor bleb) (Figure 1). Visualization of microcystic spaces was graded on descending Likert scale: 1, good number of microcysts; 2, few; and 3, nil (Figure 2).

The primary outcome parameter noted was change in IOP pre- and postoperatively. The secondary outcome parameter was alterations in the bleb morphology and bleb failure. Bleb failure was defined as IOP of > 18mm Hg or increase in the number of AGMs by ≥1 or a decrease in the bleb height by minimum of 20% at the four-month follow-up. Complete success was defined as IOP <18 mm Hg without AGMs. Qualified success was defined as IOP<18 mm Hg with AGM.

Statistical analysis

The data were entered into Microsoft Excel. Statistical Package for Social Sciences (SPSS) Version 17.0 software (IBM Corp., Armonk, NY) was used for data analysis. The ordinal data analyzed using Friedman test. The quantitative data were analyzed using the chi-square test or Mann-Whitney U test. Correlations were analyzed using the Pearson correlation coefficient. A p-value of <0.05 was considered statistically significant.

## Results

The study included 35 patients; group A comprised 17 cases, whereas group B comprised 18 cases. The baseline characteristics of each group are detailed in Table 1. The most frequently encountered intra-operative complications were related to the iris, namely floppy iris, which occurred in eight patients. One patient in the visco-cushion group with uneventful surgery presented with malignant glaucoma two months postoperatively and was medically managed. Rest of the intra- and postoperative complications are given in Table 2. Intra-operatively, a ubiquitous finding was staining of blebs by Trypan blue dye used for staining of the anterior capsule prior to capsulorrhexis, thereby re-confirming patency of the sclerostomy and functioning of bleb. All postoperative complications resolved with conservative management. None of our patients had any abnormal AC inflammation, as visible on slit-lamp examination in the postoperative period.

**Table 1:** Baseline characteristics in both groups

Criteria	Group A, N=17	Group B, N=18	Overall, N=35
Age (years), mean±SD	58.94±7.66	60.83±7.62	59.91±7.59
Gender (male:female)	8:10	8:9	16:19
Interval between trabeculectomy and phacoemulsification (years), mean±SD	7.12±5.74	6.56±5.55	6.83±5.57
Visual acuity (logMAR), mean±SD	1.36±0.37	1.39±0.39	1.38±0.38
Number of anti-glaucoma medications, mean±SD	0.71±1.1	0.39±0.7	0.54±0.92
IOP (mm Hg), mean±SD	15.71±2.91	15.17±2.38	15.43±2.62

**Table 2:** Complications of phacoemulsification in PACG patients with and without visco-cushion IOL, intraocular lens; PACG, primary angle closure glaucoma

	Group A	Group B	Total
Intra-operative			
Floppy iris	4	4	8
Vitreous loss	1	0	1
Zonular dialysis	0	1	1
IOL haptic break	1	0	1
Postoperative			
Posterior capsular opacification	1	3	4
Cystoid macular edema	1	0	1
Malignant glaucoma	0	1	1

**Figure 3:**
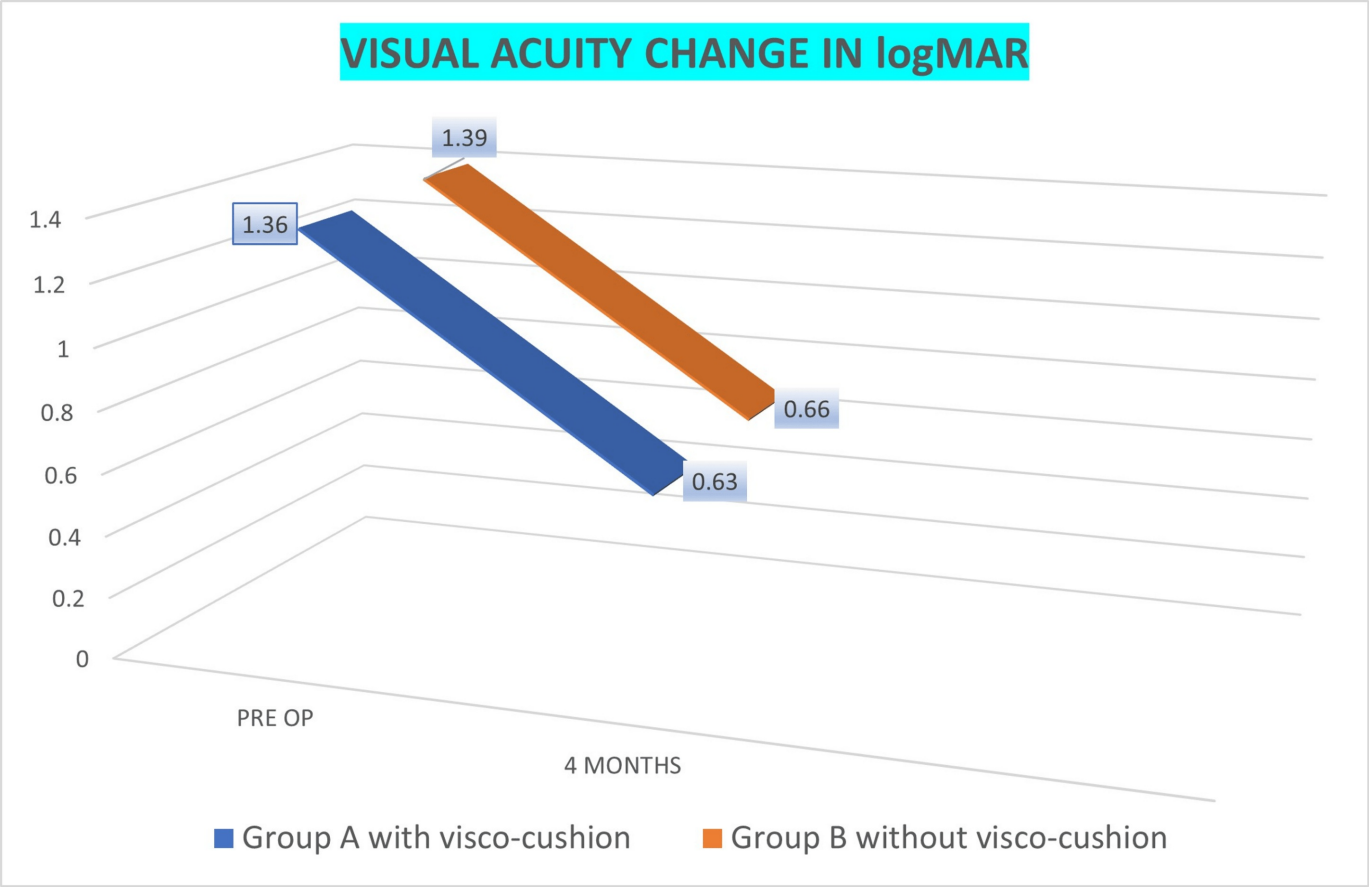
Visual acuity changes post-phacoemulsification in PACG patients with and without visco-cushion PACG, primary angle closure glaucoma

There was a mean visual acuity increase of five Snellen lines after phacoemulsification in both groups, and the mean final visual acuity at four months was LogMAR 0.60 (Figure 3). Majority of the patients had significant increase in visual acuity (13 eyes achieving acuity of LogMAR 0.30 or better), with five patients with advanced glaucomatous damage getting no visual gain.

Primary outcome

**Figure 4:**
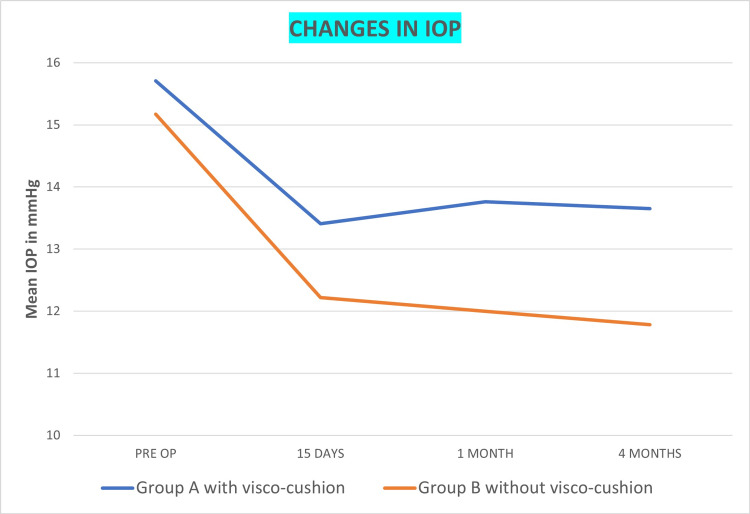
IOP changes post-phacoemulsification in PACG patients with and without visco-cushion Inter-group difference (ANOVA test): p-value=0.015 Intra-group difference (Freidman test): p-value=0.000 for both groups IOP, intraocular pressure; PACG, primary angle closure glaucoma

Overall, the IOP was 15.43 ± 2.62 mm Hg preoperatively, which decreased significantly to 12.69 ± 2.32 mm Hg at the four-month postoperative visit (p= 0.000, Friedman test) (Figure 4). Additionally, there was a significant difference in IOP reduction between the two groups, with lower IOP in group B without visco-cushion (11.78 ± 2.39 mm Hg) compared with visco-cushion group (13.65 ± 1.90 mm Hg) at the final follow-up visit (p = 0.015, ANOVA test).

Preoperatively, 23 patients were categorized as complete success and 12 as qualified success, all of whom achieved complete success at four months postoperatively except one eye presenting with malignant glaucoma on postoperative day 40, which resolved with treatment. The mean number of AGM decreased significantly in both groups (Table 3).

**Table 3:** Changes in trabeculectomy success post-phacoemulsification in PACG patients with and without visco-cushion PACG, primary angle closure glaucoma

	N	Preoperative		4 months postoperative	
		Complete success	Qualified success	Complete success	Qualified success
Group A	17	12	5	17	0
Group B	18	11	6	17	1

The correlation of IOP change to different bleb morphology parameters is given in Table 4. The change in IOP from preoperatively to four months positively correlated to preoperative IOP and negatively correlated to bleb height changes on AS-OCT.

**Table 4:** Correlation of IOP changes with bleb morphology parameters Group A + Group B, N=35 IOP, intraocular pressure; ACD, anterior chamber depth; IBAGS, Indiana Bleb Appearance Grading Scale; OCT, optical coherence tomography

Variables	P-value (Pearson correlation coefficient)
Preoperative IOP	p=0.000, r= 0.685
ACD change	p=0.756
Bleb height (IBAGS)	p=0.062
Bleb extent (IBAGS)	p=0.394
Bleb vascularity (IBAGS)	p=0.978
OCT bleb height	p=0.003, r= -0.488
OCT bleb wall thickness at 12 0’ clock	p=0.434
OCT bleb wall thickness at sclerostomy	p=0.200
OCT bleb maximum wall thickness	p=0.197
Bleb internal reflectivity	p=0.685
Microcystic spaces	p=0.250
Macrocystic spaces	p=0.729

Secondary outcomes

All blebs were evaluated using the IBAGS criteria, namely height, extent, vascularity, leakage (by Seidel’s test), and AS-OCT. We used the Friedman statistical test to better evaluate the continuous intra-group changes rather than at the final (four-month) postoperative visit alone.

IBAGS

The changes in each component of IBAGS at four months postoperative compared to preoperative status is detailed in Table 5. The intra-group variation over time is tested using the Friedman test, and the intergroup difference is analyzed using the Fischer exact test. Bleb extent did not show significant changes in either group. A statistically significant reduction in bleb height (p value= 0.041, Friedman test) and increased vascularity (p value=0.016, Friedman test) were noted in group B without a visco-cushion. However, the difference between the two groups was not significant for any parameters. Postoperatively, none of the patients presented with bleb leak, as evidenced by a negative Seidel's test.

**Table 5:** Changes in bleb morphology as graded by IBAGS post-phacoemulsification in PACG patients with and without visco-cushion *P-value calculated using the Friedman test #P-value calculated using Fischer’s exact test IBAGS, Indiana Bleb Appearance Grading Scale; PACG, primary angle closure glaucoma

IBAGS component	Group A (with visco-cushion), N=17			Group B (without visco-cushion), N=18			Intergroup difference^#^
Change noted	Reduced	Same	Increased	Reduced	Same	Increased	
Height	2	10	5	3	13	2	0.119
Intra-group variation*	0.338			0.041			
Extent	4	10	3	3	13	2	0.601
Intra-group variation*	0.965			0.779			
Vascularity	3	8	6	2	7	9	0.979
Intra-group variation*	0.104			0.016			

AS-OCT

**Figure 5:**
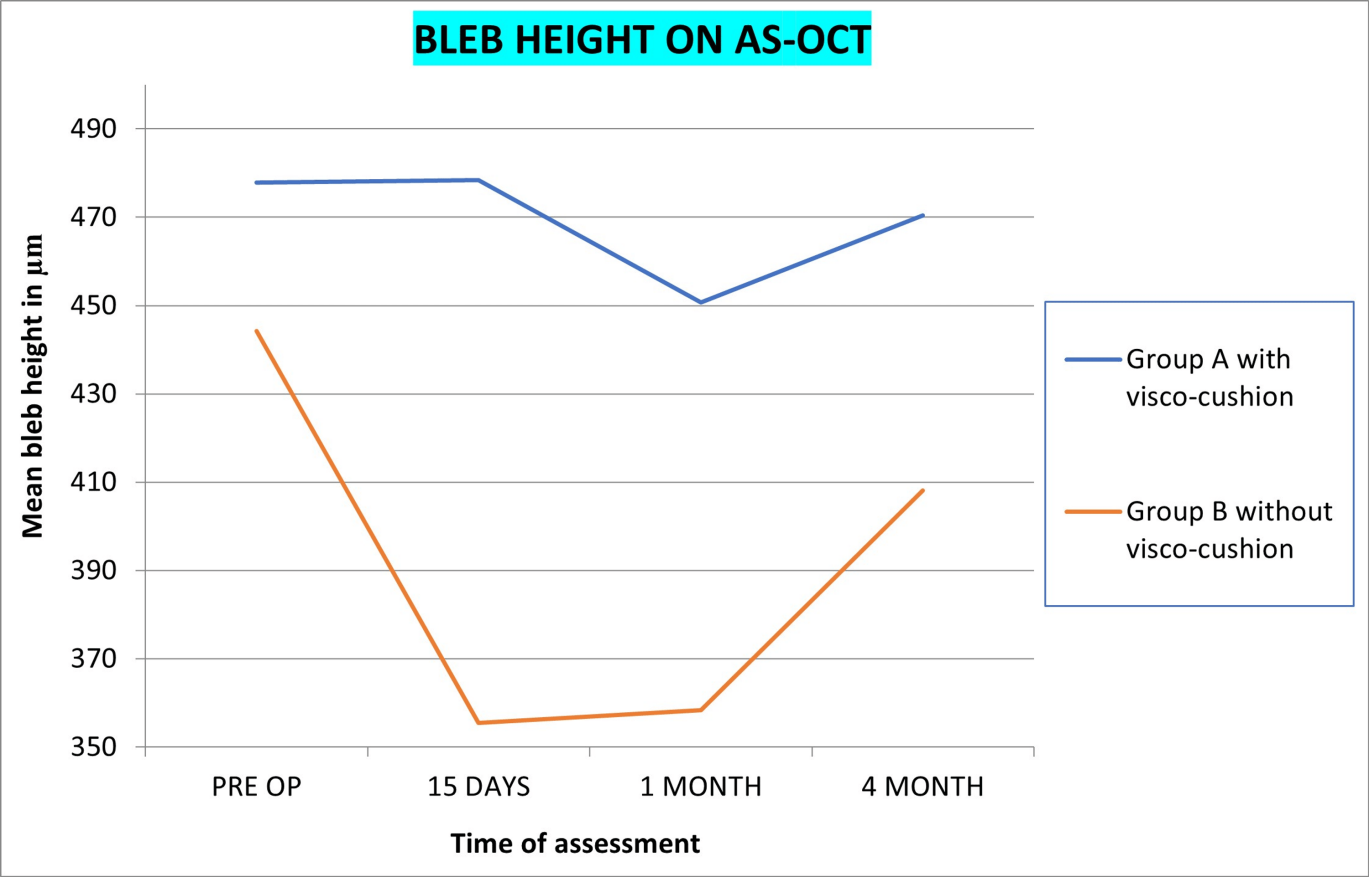
Mean bleb height changes post-phacoemulsification in PACG patients with and without visco-cushion (AS-OCT) PACG, primary angle closure glaucoma; AS-OCT, anterior segment optical coherence tomography

**Figure 6:**
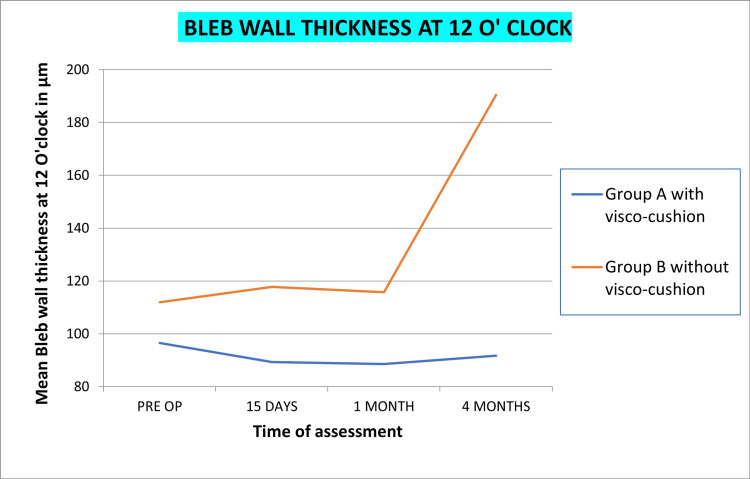
Changes in mean bleb wall thickness at the 12 O' clock position, post-phacoemulsification in PACG patients with and without visco-cushion PACG, primary angle closure glaucoma

**Figure 7:**
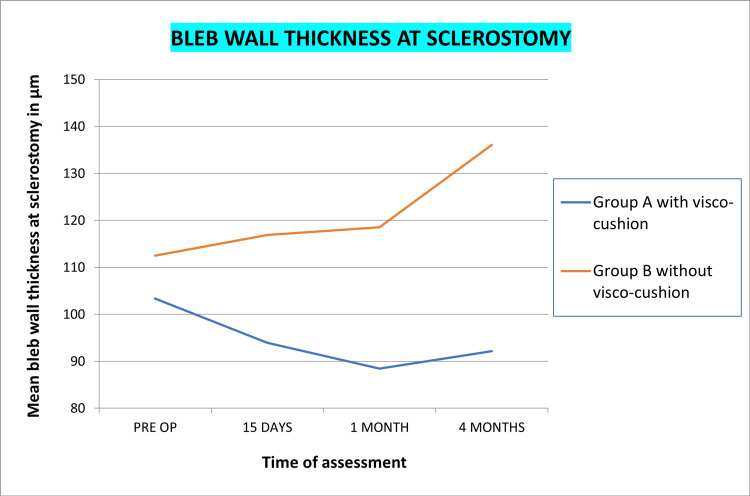
Changes in mean bleb wall thickness at sclerostomy, post-phacoemulsification in PACG patients with and without visco-cushion PACG, primary angle closure glaucoma

**Figure 8:**
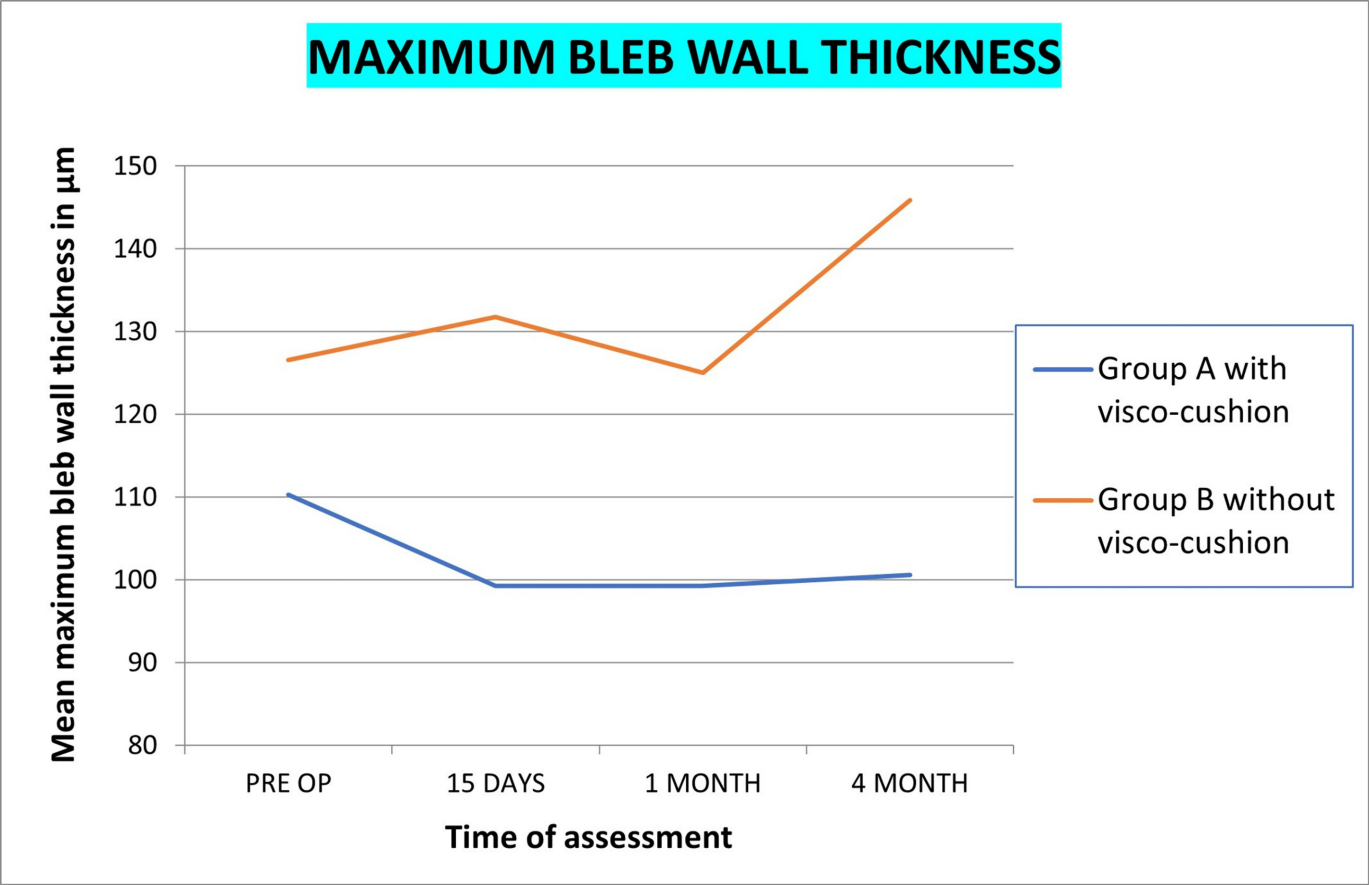
Changes in maximum bleb wall thickness, post-phacoemulsification in PACG patients with and without visco-cushion PACG, primary angle closure glaucoma

Bleb height at the highest location, bleb wall thickness at 12 O’ clock, bleb wall thickness at sclerostomy, and maximum bleb wall thickness in both groups is shown in Figures 5-8, respectively. Statistically significant reduction of bleb height and increased wall thickness was noted in group B without visco-cushion. The statistical analysis of intra-group variation from preoperative to four-month postoperative assessment and intergroup difference of these parameters at four months is compiled in Table 6.

**Table 6:** Quantitative bleb analysis by AS-OCT: statistical analysis (p-values) AS-OCT, anterior segment optical coherence tomography

	Group A intra-group variation	Group B intra-group variation	Inter-group difference
Statistical test	Friedman test	Friedman test	Mann-Whitney U test
Bleb height	0.824	0.031	0.038
Bleb wall thickness at the 12 O’clock position	0.451	0.132	0.003
Bleb wall thickness at sclerostomy	0.411	0.159	0.003
Maximum bleb wall thickness	0.142	0.245	0.001

Bleb internal reflectivity was maintained in 23 (66%) patients, which was similar in both groups. Microcystic spaces were significantly increased in group A with visco-cushion compared to the other group (p-value= 0.004, Fischer exact test). The overall changes in these parameters in both groups are shown in Table 7. Macrocystic spaces, i.e., cystic appearance of bleb, reduced from six cases to four cases in group A with visco-cushion and from four cases to two cases in group B without visco-cushion.

**Table 7:** Changes in internal reflectivity and microcystic spaces on AS-OCT, post-phacoemulsification in PACG patients with and without visco-cushion #P-value calculated using Fischer's exact test AS-OCT, anterior segment optical coherence tomography; PACG, primary angle closure glaucoma

AS-OCT component	Group A (with visco-cushion), N=17			Group B (without visco-cushion), N=18			Intergroup difference^#^
Change noted	Reduced	Same	Increased	Reduced	Same	Increased	
Internal reflectivity	4	9	4	2	14	2	0.457
Microcystic spaces	2	10	5	3	13	2	0.004

Bleb Failure

As predefined in our protocol, a total of 30 cases had bleb failure, as detailed in Table 8. However, none of these blebs failed as per IOP criteria. Difference of bleb failure rate for both groups was not statistically significant (p=0.698, chi-square test). All mature blebs (˃1 year old) were robust enough to respond similarly to the stress of phacoemulsification.

**Table 8:** Bleb failure as per each of criterion post-phacoemulsification in PACG patients with and without visco-cushion Mann-Whitney U test p-value=0.803 AS-OCT, anterior segment optical coherence tomography; IOP, intraocular pressure; PACG, primary angle closure glaucoma

Criterion	Group A with visco-cushion	Group B without visco-cushion
IOP ˃ 21 mm Hg	0	0
Requirement of ≥1 additional anti-glaucoma medication	0	1
Bleb height reduction by at least 20% on AS-OCT	14	16

## Discussion

Cataract extraction in itself has been found to reduce IOP by 1.5 to 2 mm Hg in non-glaucomatous eyes proportionate to preoperative IOP and shallowness of AC [17,18]. For glaucomatous eyes, this reduction has been documented to be higher (2-4.5 mm Hg) in POAG cases [19] and maximum in PACG cases [20-22] (4-6 mm Hg). Few studies have reported extreme effects of cataract in unresolved acute angle closure glaucoma with IOP as high as 55 mm Hg [23]. This IOP reduction post-cataract extraction is usually a short-term response [20,21,23-27]. A study by Tham et al. confirmed this short-term effect by reporting requirement of trabeculectomy in 17.7% of PACG cases primarily treated with phacoemulsification after a period of two years [28].

Ocular viscoelastic devices (OVDs) have been in use since the 1970s for improving cataract surgery outcomes [29]. Viscocohesive substances are long chain molecules that coalesce with each other and are used in cases of shallow AC and elastic anterior capsule (pediatric cataracts), where they help by increasing working space and flattening anterior capsule, thereby facilitating capsulorrhexis. These OVDs can be washed out of AC in bolus form since molecules adhere to each other. They can clog trabecular meshwork and raise postoperative IOP in few eyes [30]. Healon GV® from Abott Laboratories is a cohesive viscoelastic substance with superhigh viscosity frequently used in situations of difficult capsulorrhexis. Further exploiting its features, we tried using Healon GV® to block sclerostomy ostium during phacoemulsification. We hypothesized that blocking the sclerostomy would reduce aqueous turbulence in blebs, thereby preventing the release of inflammatory mediators following cataract surgery and bleb failure. To the best of our knowledge, this modality of safeguarding bleb has not been documented in the literature.

Primary outcome: quantitative changes in IOP

Therapy for all subtypes of glaucoma primarily aim at reducing IOP, including normal tension glaucoma, with further lowering of IOP being protective. IOP plays a primary role in both deciding management protocols and evaluating efficacy of any treatment. In our study, mean IOP reduction of 17% at 15 days after phacoemulsification was maintained till four months. This statistically highly significant reduction in IOP was higher for group B without visco-cushion.

Mechanism of action for control of IOP post-phacoemulsification has been postulated to be release of pupillary block [31], widening of AC angle [31], reduction in extent of peripheral anterior synechiae [32], and enhanced uveoscleral outflow [11]. Additionally, the change in force exerted on ciliary body due to capsular bag contraction after phacoemulsification results in greater reduction of aqueous production in eyes with preoperative shallow AC [17,31]. Poley et al. suggested that lens removal allows posterior capsule to move posteriorly, dislodging zonules over ciliary body, with a consequent widening of Schlemm’s canal and improvement in aqueous humor drainage [27,33]. Another proposed mechanism states that ultrasound used during phacoemulsification procedure is responsible for an abrupt rise in the AC pressure, producing inflammatory cytokines (mostly IL-1), which stimulate metalloproteinase production and trabecular meshwork remodeling, thereby facilitating aqueous humor drainage [33,34].

In the current study, phacoemulsification in eyes with functional filters resulted in further opening of angles. Improved aqueous drainage contributed to improved IOP control (IOP reduction of 2.7mm Hg at four months). This finding has been confirmed by Moghimi et al., who also documented an IOP reduction of 3 mm Hg over a one-year period after phacoemulsification concomitant with significant requirement in the use of AGMs [31]. Khokhar et al., on the other hand, could not document any significant IOP reduction at any time post-phacoemulsification up to a follow-up of six months [35].

Although bleb morphology was better preserved with visco-cushioning, IOP reduction was less pronounced in this group, suggesting a potential trade-off between structural integrity and pressure control. The reduction in IOP post-cataract surgery has been documented to be proportionate to both preoperative IOP and quantitative changes in bleb height [20-22]. Our study confirmed this positive correlation with preoperative IOP (p=0.000, r=0.685). Therefore, we conclude that eyes with higher IOP before cataract surgery had a more robust response with IOP reduction.

Secondary outcome: bleb morphology changes

Twenty-one (60%) blebs depicted no change in bleb extent, which was similar in both groups. Most other studies have documented a bleb extent compromise in almost three-fourths of patients [2,5,11]. One study reported bleb scarring in 35% cases, along with complete loss of bleb function in two cases requiring repeat trabeculectomy [36]. One study has also advocated simultaneous internal revision of bleb employed during phacoemulsification as a prophylactic measure to prevent bleb scarring [37].

Around 23 (66%) cases had maintained preoperative bleb height, of which eight cases had a transient reduction in the immediate postoperative period. This could be due to transiently reduced aqueous outflow through sclerostomy secondary to increased uveoscleral outflow induced by inflammatory mediators, as postulated by Klink et al. [6]. An increased bleb height was documented in seven (20%) cases and reduction in five (14%) cases, with the changes being significant in group B without visco-cushion but not in group A with visco-cushion implying beneficial effect of visco-cushion on bleb morphology protection. Previous studies have differed with us in reporting an almost ubiquitous finding of reduction in bleb height post-phacoemulsification in open angle glaucoma cases [2,5,6,11,36].

**Figure 9:**
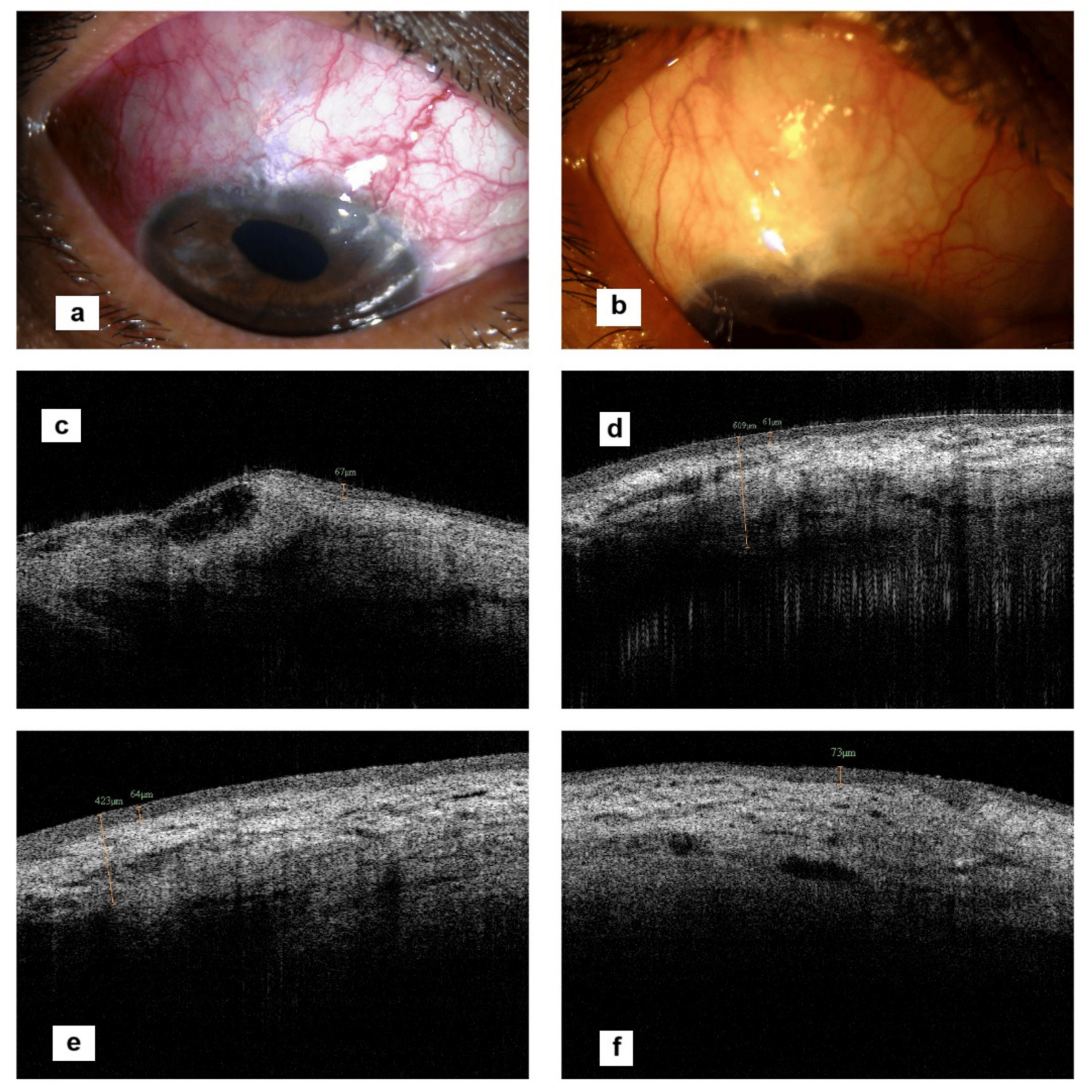
A patient of group B without visco-cushion showing transient increase in vascularity and internal reflectivity of bleb at 15 days Figure 9a showing E1H1V3 bleb at postoperative day 15 along with an increased internal reflectivity on AS-OCT (Figure 9d) compared to preoperative AS-OCT (Figure 9c). At four months, the bleb improved to E2H2V1 (Figure 9b) with decreased internal reflectivity and appearance of microcystic spaces starting from one-month postoperative period (Figure 9e), with a good morphology restoration by month 4 (Figure 9f).

Bleb vascularity indicates aggressive healing and/or persisting inflammation leading to collagen formation, shrinkage of which causes vessel corkscrewing. If prolonged, increased vascularity contributes to scarring and bleb failure. Increased vascularity post-phacoemulsification reflects increased permeability of blood-aqueous barrier, inducing an inflammatory process [2]. Our study documented 15 (43%) blebs with maintained vascularity, of which two blebs manifested only transient increase (Figure 9) and five (14%) blebs showed decreased vascularity. Around 15 (43%) blebs presented with increased vascularity. This increased vascularity could reflect subclinical inflammation and/or scarring, not evident by AC inflammation. A similar finding has been reported by previous studies [5,6]. In group sans viscoelastic protection of sclerostomy site (B), this increased vascularity was significant (nine cases) compared with group A with viscoelastic protection of sclerostomy site (six cases). This implies beneficial effect of visco-cushion on subclinical intra-bleb inflammation.

Quantitative changes by AS-OCT

**Figure 10:**
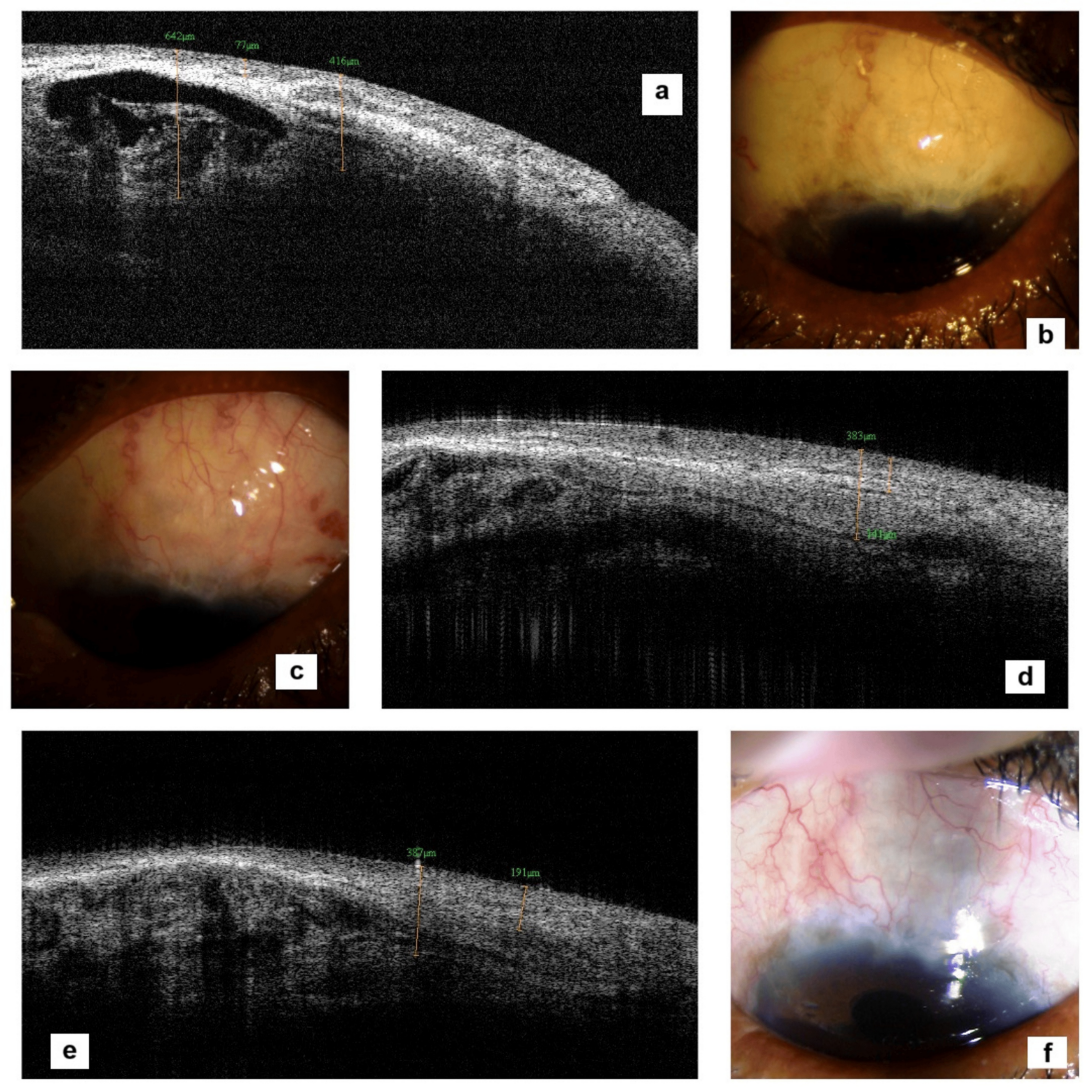
Qualitative and quantitative changes in bleb morphology post-phacoemulsification with visco-cushion Figure 10a and Figure 10b shows preoperative E2V1H3 bleb with macrocystic spaces on AS-OCT, respectively. Figure 10c and Figure 10d show mild decrease in bleb height, increased vascularity, and internal reflectivity one month after phacoemulsification, with all the parameters improving to baseline by month 4. Figure 10e shows maintained bleb morphology by IBAGS, and Figure 10f shows replacement of macrocyst to microcystic spaces on AS-OCT. This patient’s status improved from qualified success to complete success post-phacoemulsification.

Although bleb height reduction was noted in both the groups, the reduction was significant in group B without visco-cushion. Maximum reduction was noted immediately after surgery until one month followed by slow recovery by the fourth month. In group A with visco-cushion, the timeline differed in that bleb height was maintained at 15 days postoperative, decreased slightly at one month, and again recovered to previous values at four months (Figure 10). Thus, visco-cushion blunted the response of phacoemulsification on bleb height. The qualitative assessment data on IBAGS height also suggests the same.

Bleb wall thickness was measured at three points: at sclerostomy site, at the 12 o’clock position, and at area with maximum thickness. Alteration in wall thickness at all three points, for both groups, was statistically insignificant. The intergroup difference (group A and B) however achieved statistical significance, with greater thickening being noted in group B without visco-cushion. Thickening of bleb wall has been linked to better functionality [37-39]. Visco-cushioning led to maintained bleb height, lower internal reflectivity, and increased microcystic spaces as compared to group B without visco-cushion. However, higher bleb wall thickness was noted in group B without visco-cushion, probably attributed to a greater reduction in bleb height/compaction of the bleb. Although both groups achieved a significant IOP reduction, the transient effect on IOP reduction was more significant in the non-visco-cushion group, likely in agreement with the change in bleb wall thickness. Thus, our results suggest that bleb wall thickness is the most important determinant of bleb function.

The internal reflectivity of bleb on AS-OCT is the counterpart of vascularity in IBAGS. We evaluated internal reflectivity by a subjective assessment, as no standardized grading of reflectivity has been proposed. We found 66% (23/35) blebs to retain their preoperative reflectivity, of which seven patients had a transiently hyperreflectivity in the immediate postoperative period. As explained by Fakhraie et al., this could be a result of transient increase in vascularity [40]. Internal reflectivity is indirect evidence of intra-bleb inflammation/scarring, and low reflectivity has long been regarded as a sign of good bleb function [37,40,41]. Hence, we conclude that a good bleb function was retained in 66% cases and improved function in 17% of blebs.

Microcystic spaces represent the degree of transconjunctival aqueous flow and have been correlated with functioning of blebs [37,38,42]. While an adequate number of microcysts were maintained in 66% (23/35) cases, microcystic spaces significantly increased in group A with visco-cushion, thus emphasizing bleb protective effect of visco-cushion. The macrocystic spaces visible in 10 blebs preoperatively decreased in size postoperatively and disappeared completely in six cases independent of the use of visco-cushion. All these 10 blebs still had an adequate number of microcysts with gradual increase in number, indicating retention of bleb function.

The protective effect of visco-cushion on bleb morphology paradoxically fails to resonate with sustenance of bleb function. Clinicians may need to weigh the benefits of structural preservation against the primary goal of IOP control. However, the changes in bleb morphology evolve over months, and protective effect of visco-cushion on bleb morphology possibly could translate into better long-term IOP control and survival of these blebs. Long-term follow-up of these cases is suggested to verify this conjecture.

The strength of this study was a matched control group, with all confounders of surgical outcomes accounted for. The objective bleb evaluation by AS-OCT parameters and clinically by IBAGS reduced the observer bias to a minimum. The major limitation of this study would probably be the intermediate period of follow-up of four months, wherein the changes in bleb morphology were probably still in transition; therefore, we recommend a longer assessment of these blebs over one to two years post-phacoemulsification. Also, a bigger sample size would help draw more affirmative conclusions about the changes in bleb morphology in the two groups and their long-term impact on IOP control.

## Conclusions

Phacoemulsification in filtered PACG eyes with advanced optic nerve damage yielded good visual recovery. Phacoemulsification in PACG eyes had a beneficial effect on IOP control with additional IOP reduction of 2.7 mm Hg, with blunting of this effect by the use of visco-cushion. Phacoemulsification induces significant bleb height attenuation, increased bleb vascularity, and reduction in microcystic spaces. Intra-operative use of highly cohesive viscoelastic cushion to block sclerostomy ostium can be considered for preserving bleb morphology post-phacoemulsification with minimal compromise of IOP lowering effect for short-term. Bleb failure signs can be subtle, with reduction of bleb height seen in 86% cases, and needs to be monitored over time after cataract surgery, with less reliance on IOP. To summarize, while visco-cushioning preserves trabeculectomy bleb morphology after phacoemulsification, its impact on IOP control is limited, necessitating long-term studies to evaluate its clinical utility. We do not recommend viscoelastic blocking of internal sclerostomy during phacoemulsification based on our short-term study results.
