# Correction: Impact of Phacoemulsification on Trabeculectomy Bleb Function and Morphology in Primary Angle Closure Glaucoma: A Comparative Study of the Visco-Cushion Effect

**DOI:** 10.7759/cureus.c281

**Published:** 2025-09-04

**Authors:** Kirti Singh, Keerti Wali, Arshi Singh, Mainak Bhattacharyya, Sonal Dangda

**Affiliations:** 1 Ophthalmology, Guru Nanak Eye Centre, Delhi, IND; 2 Ophthalmology, Shri B. M. Patil Medical College, Bijapur Lingayat Development Education (BLDE) (Deemed to be University), Vijayapura, IND; 3 Ophthalmology, EyeQ & Max Healthcare, Ghaziabad, IND; 4 Ophthalmology, Atrium Health Wake Forest Baptist, Winston-Salem, USA

This article's ethics statement has been corrected to include the approval number (F.No/11/IEC/MAMC/2011/147), which was erroneously omitted from the initial submission.

